# Supplementary material for: Hyperphosphatemia increases inflammation to exacerbate anemia and skeletal muscle wasting independently of FGF23-FGFR4 signaling
Source: eLife. 2022 Mar 18;11:e74782. doi: 10.7554/eLife.74782 (PMC8963881; doi:10.7554/eLife.74782)

Figure 6-source data 1

A

As shown in manuscript

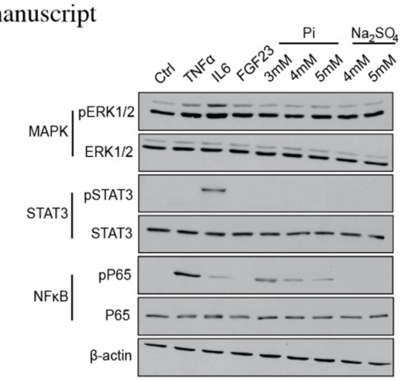

Raw immunoblots

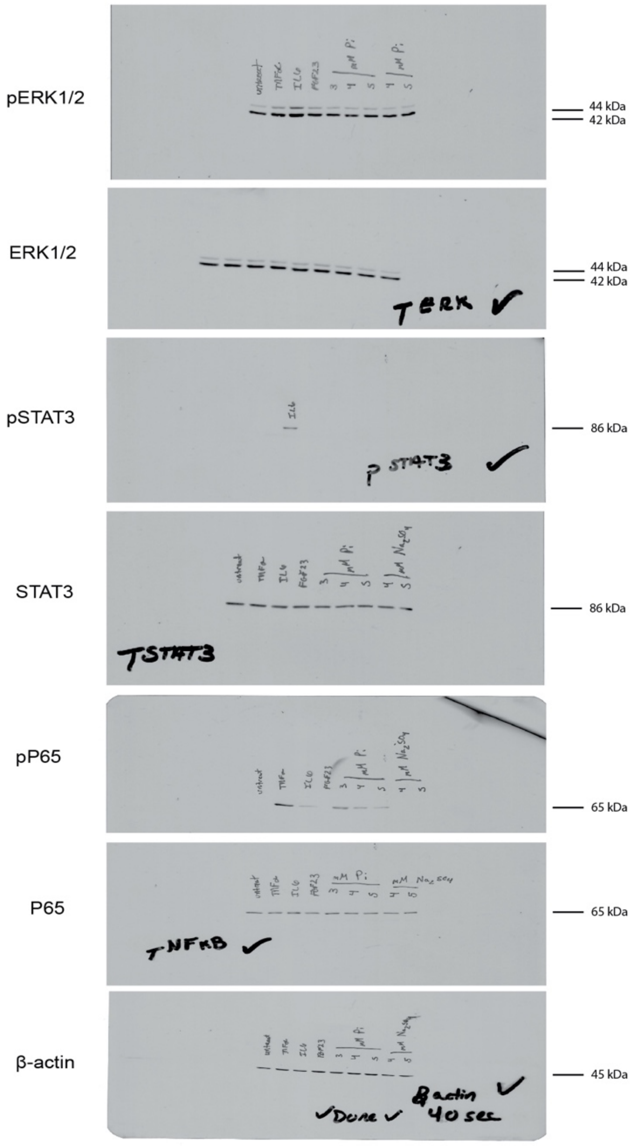

G

As shown in manuscript

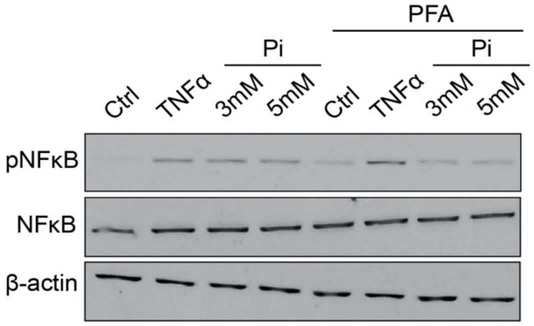

Raw immunoblots

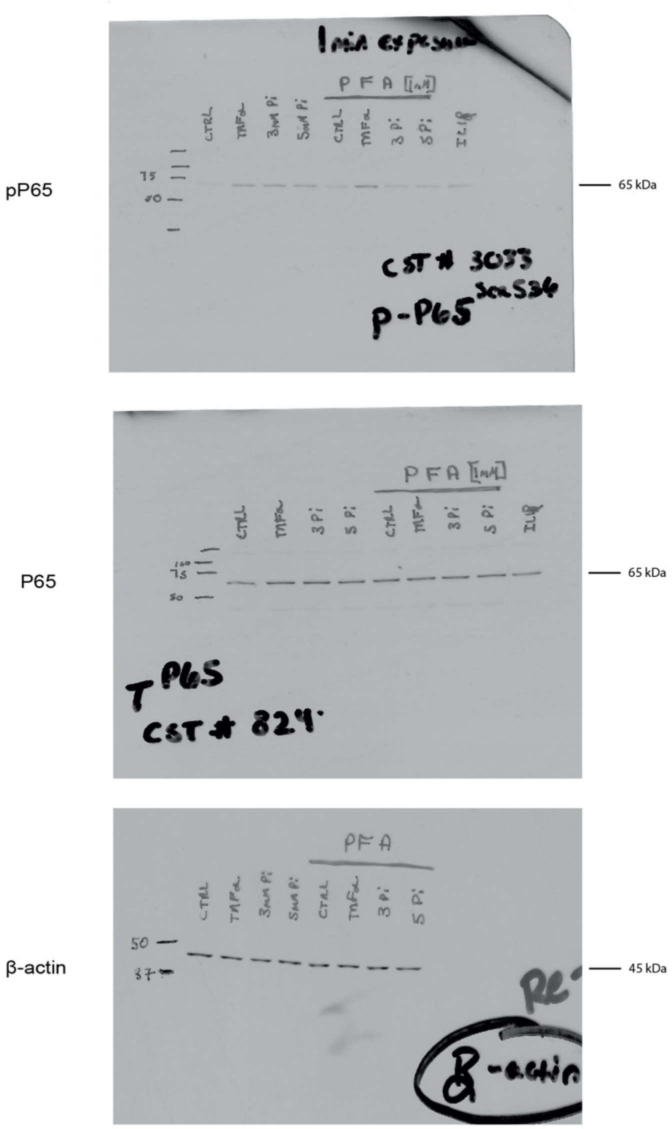

Supplement: Figure 6—source data 1. — Original uncropped western blots of the cropped western blots shown in Figure 6A, G. The molecular weight is indicated on the right in kDa. [file elife-74782-fig6-data1.zip › Figure 6AG source data_rev.pdf]
